# Assessing the Accuracy of Smartwatch-Based Estimation of Maximum Oxygen Uptake Using the Apple Watch Series 7: Validation Study

**DOI:** 10.2196/59459

**Published:** 2024-07-31

**Authors:** Polona Caserman, Sungsoo Yum, Stefan Göbel, Andreas Reif, Silke Matura

**Affiliations:** 1 Serious Games Research Group Technical University of Darmstadt Darmstadt Germany; 2 Department of Psychiatry, Psychosomatic Medicine and Psychotherapy Goethe University Frankfurt University Hospital Frankfurt am Main Germany

**Keywords:** maximal oxygen uptake, oxygen consumption, cardiorespiratory fitness, physical fitness, physical activity, fitness tracker, wearables, wearable, exercise, fitness, tracker, trackers, cardiorespiratory, wrist worn device, devices, validation study, VO2max, sport watch, fitness level, mobile phone

## Abstract

**Background:**

Determining maximum oxygen uptake (VO_2_max) is essential for evaluating cardiorespiratory fitness. While laboratory-based testing is considered the gold standard, sports watches or fitness trackers offer a convenient alternative. However, despite the high number of wrist-worn devices, there is a lack of scientific validation for VO_2_max estimation outside the laboratory setting.

**Objective:**

This study aims to compare the Apple Watch Series 7’s performance against the gold standard in VO_2_max estimation and Apple’s validation findings.

**Methods:**

A total of 19 participants (7 female and 12 male), aged 18 to 63 (mean 28.42, SD 11.43) years were included in the validation study. VO_2_max for all participants was determined in a controlled laboratory environment using a metabolic gas analyzer. Thereby, they completed a graded exercise test on a cycle ergometer until reaching subjective exhaustion. This value was then compared with the estimated VO_2_max value from the Apple Watch, which was calculated after wearing the watch for at least 2 consecutive days and measured directly after an outdoor running test.

**Results:**

The measured VO_2_max (mean 45.88, SD 9.42 mL/kg/minute) in the laboratory setting was significantly higher than the predicted VO_2_max (mean 41.37, SD 6.5 mL/kg/minute) from the Apple Watch (*t*_18_=2.51; *P*=.01) with a medium effect size (Hedges *g*=0.53). The Bland-Altman analysis revealed a good overall agreement between both measurements. However, the intraclass correlation coefficient ICC(2,1)=0.47 (95% CI 0.06-0.75) indicated poor reliability. The mean absolute percentage error between the predicted and the actual VO_2_max was 15.79%, while the root mean square error was 8.85 mL/kg/minute. The analysis further revealed higher accuracy when focusing on participants with good fitness levels (mean absolute percentage error=14.59%; root-mean-square error=7.22 ml/kg/minute; ICC(2,1)=0.60 95% CI 0.09-0.87).

**Conclusions:**

Similar to other smartwatches, the Apple Watch also overestimates or underestimates the VO_2_max in individuals with poor or excellent fitness levels, respectively. Assessing the accuracy and reliability of the Apple Watch’s VO_2_max estimation is crucial for determining its suitability as an alternative to laboratory testing. The findings of this study will apprise researchers, physical training professionals, and end users of wearable technology, thereby enhancing the knowledge base and practical application of such devices in assessing cardiorespiratory fitness parameters.

## Introduction

The concept of the maximum oxygen uptake (VO_2_max), established in 1923 by Hill and Lupton [1] is a fundamental measure in assessing cardiorespiratory fitness [2] and is also often used to determine an individual’s physical fitness level [3,4]. Cardiorespiratory fitness is defined as the ability of the circulatory and respiratory systems to supply oxygen to the muscles during sustained physical activity [3]. VO_2_max is also often used as a performance measure [5,6]. Previous research concludes that VO_2_max is closely related to all-cause mortality and underscores the importance of enhancing VO_2_max to reduce the risks of developing cardiovascular diseases [7-10].

Typically, VO_2_max is measured in a controlled laboratory setting using a metabolic gas analyzer during an incremental exercise test, commonly administered on a motorized treadmill or a cycle ergometer [7]. During the test, either the speed on the treadmill or the resistance on the ergometer is gradually increased, until participants reach maximum exhaustion. Such tests are typically directed toward special populations, for example, individuals with known or suspected cardiovascular diseases or endurance athletes. Laboratory tests require expensive equipment (ie, a metabolic gas analyzer) and trained personnel and are therefore often costly and time-consuming. As the maximal exercise test necessitates participants to achieve maximal exertion, it may not always be safe for everyone, especially not without medical supervision and emergency equipment [11]. Accordingly, given the impracticality of VO_2_max assessments for everyday application and their limited accessibility by the general population, the emergence of fitness trackers has provided a convenient and accessible alternative for estimating VO_2_max in real-world settings. A recent survey shows that 21% of Americans already use a smartwatch or a fitness tracker such as the Garmin, Fitbit, or Apple Watch [12]. According to another recent survey, wearable technology has also been identified as the number one fitness trend in 2022 [13].

Prior investigations have already assessed the reliability and validity of various wearables, using heart rate (HR) as a metric for quantifying individual physiological exertion [14]. Further studies have explored the potential of biometric monitoring technologies in estimating users’ cardiovascular fitness levels, using algorithms like those developed by Firstbeat Analytics [15] and used by prominent brands such as Garmin and Huawei [16]. Additionally, researchers developed their methodologies to calculate oxygen uptake using wearable devices or smartphones [17-20]. Previous research further validated various fitness tests carried out using smartphones, offering additional insights into the accuracy of these devices in evaluating physical metrics [21,22]. Despite the promising potential of wrist-worn devices in facilitating fitness assessments, concerns have been raised regarding the accuracy and reliability of estimating parameters, such as VO_2_max or VO_2_ peak, with particular concern about their potential misuse by consumers for making medical decisions [23]. While several studies have shown that wearables are very accurate [15,24-29], contradictory evidence suggests potential overestimation or underestimation in VO_2_max measurements [30-33]. Notably, only little research has been conducted on the accuracy of VO_2_max predictions among participants with varying fitness levels, particularly those with lower or higher fitness levels [34,35].

Given the Apple Watch’s dominant position in the global smartwatch market with the largest share of shipments [36] and being the primary choice for the majority of users [12], assessing the accuracy and reliability of its VO_2_max estimation becomes critical in determining its potential as a dependable alternative to traditional laboratory testing. However, only a little research has been conducted evaluating the accuracy of the Apple Watch in estimating cardiorespiratory fitness indicators. Most of the studies that validated the accuracy of the Apple Watch focused on fitness parameters such as energy expenditure, HR, HR variability, or oxygen consumption reserve [37-41]. There remains a gap in the literature regarding the specific evaluation of the Apple Watch to predict VO_2_max. While Apple has conducted an extensive study to validate its VO_2_max estimation algorithm [42], concerns exist regarding potential bias and the limited medical representativeness of their findings.

To address these concerns and contribute to the understanding of wearable technology in fitness assessment, this study aims to assess the accuracy and reliability of VO_2_max estimation using the Apple Watch Series 7. Toward this end, we conducted a comparative analysis between the VO_2_max estimation of the Apple Watch 7 and the gold-standard testing in a laboratory setting, using a metabolic gas analyzer. The level of agreement was evaluated using Bland-Altman plots. We calculated the error in terms of mean absolute percentage error (MAPE) and root-mean-square error (RMSE), and further assessed the reliability by calculating the intraclass correlation coefficient (ICC). The outcomes of this study will hopefully provide valuable insights into the performance of the Apple Watch Series 7 relative to other validation studies of wrist-worn devices and Apple’s validation results.

## Methods

### Ethical Considerations

The study was conducted in accordance with the Declaration of Helsinki, and approved by the Ethics Committee of the Technical University of Darmstadt (approval EK 11/2023; March 20, 2023). In the first session, all participants were informed about the specific purpose of the study. We informed them that all collected data are confidential and solely used in anonymized form. To ensure anonymity, each participant was assigned a pseudonym. Participants were informed about the risks and their right to terminate the experiment at any point without the need for an explanation. Afterward, participants provided written informed consent, completed a demographics questionnaire, and responded to inquiries regarding their physical activity.

### Study Design

The study used a repeated measures design with each participant completing 2 sessions on separate days, with a minimum resting period of 48 hours in between. Before undergoing the tests, participants were advised to refrain from consuming alcohol or any other substances that could potentially influence their respiratory system and HR. This precautionary measure aimed to ensure accurate readings and mitigate the risk of any potential false results during the testing procedure. The initial session was conducted in a controlled laboratory setting to establish a reference value for VO_2_max. The subsequent session took place on the university’s stadium track field, using the Apple Watch Series 7 to obtain an estimated VO_2_max value. Following the completion of both sessions, the VO_2_max values obtained from the 2 methods were compared against each other for analysis.

### Measurement of VO2max in a Laboratory Setting—Cycle Test

The performance test in the laboratory setting was assessed through an endurance test using a cycle ergometer. Such tests are widely used in sports science to measure VO_2_max, serving as a crucial indicator of aerobic endurance performance [43]. Due to the lack of medical expertise to conduct a maximal exercise test, we alternatively conducted a graded exercise test until subjective exhaustion. This decision was influenced by our ability to adhere to a rigorous protocol within the controlled environment of the laboratory, as well as the availability of the necessary equipment to monitor respiratory parameters and promptly terminate the session if the participant’s safety was compromised. Submaximal exercise prediction was also used in the field test using the Apple Watch, which facilitates comparison of the values derived from sessions 1 and 2.

Accordingly, the reference VO_2_max value was determined through a graded exercise test conducted on a cycle ergometer, using the portable metabolic gas analyzer (VO2 Master Health Sensors Inc [44]). Evidence of the measurement accuracy of the hardware used can be found in references [45,46]. The gas analyzer was calibrated prior to each test (ie, for each participant), using a 3-L syringe for both flow and gas calibration. Furthermore, the supervisor entered the participants’ age, sex, height, and weight in the VO2 Master Manager app (installed on an iPhone 13 Mini), which was paired with the gas analyzer. After the calibration, participants put on the electrocardiogram chest strap (Polar H10 Heart Rate Sensor [47]) and the gas analyzer while the supervisor (SY) checked the plausibility of the system (ie, both sensors connected to the smartphone via Bluetooth and transmitting the data via VO2 Master Manager app). Once participants successfully put on the equipment, they were instructed to sit on the cycle ergometer (ERGO-FIT Cycle 4073 [48]) after adjusting the seat height according to their height.

Once the setup was completed, the endurance test was conducted. The laboratory protocol was equal for male and female participants. Throughout the test, vital parameters (ie, the HR and breathing) and the participant’s current state were continuously monitored. Participants started with a 3-minute warm-up phase, riding on the cycle ergometer at a workload of 50 W at a speed of 60 rotations per minute. Afterward, the ergometer’s resistance was increased by 50 W every 2 minutes until one of the termination criteria was met (based on the criteria by Klingenheben et al [49]):

- Maximum HR, based on age and sex, individually calculated for each participant using the Fairbarn equation [50], was exceeded for 10 consecutive seconds:

HR_maxFairban_=208–0.8×age, for male participants

HR_maxFairban_=201–0.6×age, for female participants

We intentionally used the Fairbarn equation to predict the maximum HR, instead of using the Fox equation HR_maxFox_=220–age [51], which is only dependent on age. According to the analysis by Cleary et al [52], the Fairbarn equation, which considers the age and sex of the participants, is more accurate.

- Inability to maintain a pedal rate of 60 rotations per minute for more than 3 seconds
- An abnormally rapid acceleration or deceleration in HR that is not consistent with physiological norms
- Plateau in VO2, despite increasing resistance on the ergometer (increase <1 mL/kg/minute)
- Symptoms of angina pectoris (ie, pain behind the breastbone, tightness, numbness, nausea, vomiting, sweating, and shortness of breath, and anxiety)
- Other conspicuous findings, such as malaise, dizziness, headache, conspicuous pallor, and other complaints
- Signs of respiratory insufficiency could be observed, that is, participants’ ventilation reached a dangerous level (around 150 L/minute) in the VO2 Master Manager app
- Self-reported volitional exhaustion or fatigue
- Failure of monitoring equipment

At the end of the session, protocol outcomes were saved for each participant. In addition to VO_2_max, the gas analyzer provided the following parameters in real time:

- Metabolism:
  Absolute oxygen consumption (VO2 [mL/minute])
  Oxygen consumption relative to weight (VO2 [mL/kg/minute])
  Energy expenditure (Kcal/day)
  Calories (kcal/hour)
- Pulmonary function:
  Ventilation; air moved by lungs (Ve [L/minute])
  Respiratory frequency; breaths per minute (beats per minute)
  Tidal volume; volume breathed in a breath (L)
- Respiratory efficiency:
  (Ve/VO2)
  Fraction of oxygen in expired breath (FeO2 [%])
- Cardiac function:
  HR (beats per minute)
  RR Intervals (RR [milliseconds])

### Estimation of VO2max Using the Apple Watch—Track Field Test

Within 1 week after the initial laboratory session, participants were provided with an iPhone SE 2020 and an Apple Watch (Series 7, 41 mm). The Apple Watch was paired with an iPhone that had been reset to factory setting to ensure data privacy. To complete the setup of the Apple Watch, the supervisor (SY) ensured that participants entered their age, sex, height, and weight in the iPhone.

Participants were instructed to wear the Apple Watch continuously, including during sleep and showers, for at least 48 hours prior to the second session. This prolonged wearing duration was essential as the Apple Watch required at least 24 hours of continuous wear time to reliably estimate VO_2_max. The precise algorithm for VO_2_max estimation is not publicly disclosed; however, discussions with Apple technical support revealed that it incorporates resting HR measurements, exercise HR measurements, and GPS-derived velocity data from outdoor runs. To ensure a valid VO_2_max from the Apple Watch, we consulted with the manufacturer and adhered to the following procedure: participants needed to complete at least 1 training prior to the track field test, that is, an outdoor walk for 15-20 minutes. They needed to manually measure the HR every hour (using the preinstalled Health app), in addition to the passive measurements of the Apple Watch itself. Throughout the process, participants needed to ensure that the Apple Watch was always connected to the iPhone, which maintained an internet connection.

Only participants who followed the instructions and completed the outdoor walk were permitted to proceed with the run test. The run test was conducted at the university stadium at the Technical University of Darmstadt. Consistent with our laboratory protocol, we used a submaximal exercise test to mitigate the risk of injury; however, in this session, the test was conducted outdoors. The outdoor setting was necessary to ensure a sufficient GPS signal.

Before the run test, participants were given brief instructions. Particularly, they were instructed to activate the outdoor running app on their Apple Watch prior to starting the track run. To minimize the risk of injury, the protocol included a 5-minute warm-up phase, during which participants ran at a moderate pace. Following the warm-up, participants continued at a self-selected running pace, ensuring a minimum duration of 15 minutes. Once participants completed the run and returned to the starting point, they stopped the recording on their Apple Watch and proceeded with a cool-down phase. Subsequently, the supervisor accessed relevant metrics from the Health app on the paired iPhone, specifically the estimated VO_2_max in the cardio fitness section.

### Recruitment

Participants were recruited among students and employees of the Technical University of Darmstadt through the Discord server from the IT department and the university’s mailing list. To ensure a diverse range of fitness levels, we also recruited members of a local fitness studio. Eligibility criteria required participants to be older than 18 years and in good health. To streamline the selection process, the Physical Activity Readiness Questionnaire [53] was administered. As a result, individuals with any preexisting heart disease, cardiovascular conditions, orthopedic injuries, or current use of medication were deemed ineligible for participation.

To determine the required sample size, we conducted a priori power analysis using G*Power (version 3.1; Heinrich-Heine-Universität Düsseldorf) [54] with a power of 0.8, a significance level of 0.05, and a medium effect size of 0.5. This analysis indicated a minimum sample size of 27 participants. Therefore, considering expected dropouts, we initially aimed for a larger sample size of at least 30 participants. Recruitment took place over a 4-week period in the spring of 2023.

### Statistical Analysis

All data were analyzed using MATLAB (MathWorks, Inc), including external code [55,56].

We first assessed the limit of agreement between the values obtained from laboratory measurements and those provided by the Apple Watch using the Bland-Altman plot. The Bland-Altman plot enables us to evaluate if the 2 methods of measurement show a sufficient level of agreement [57]. It displays the limits of agreement by using the mean and SD of the differences between the 2 methods. As recommended by the authors themselves, 95% of the data points should lie within ±2 SD of the mean difference [57,58]. Additionally, the plot also allows us to spot outliers and to see whether there is any trend in overestimating or underestimating.

Second, in addition to the Bland-Altman plots, we calculated the ICC(2,1) to test for bias and absolute agreement in VO_2_max estimation. ICC is different from correlations such as Pearson or Spearman correlation. Calculating correlation is not appropriate to evaluate the measure of agreement, especially as the correlation coefficient depends on both the variation between individuals (ie, between the true values) and the variation within individuals (measurement error) [57]. ICC is suitable for reliability analyses, where a value less than 0.5, between 0.5 and 0.75, between 0.75 and 0.9, and greater than 0.90 indicate poor, moderate, good, and excellent reliability, respectively [59].

Third, similar to other validation studies, we used the MAPE and RMSE to calculate the overall measurement error between the VO_2_max value derived from the Apple Watch and the metabolic gas analyzer. MAPE was calculated as the average absolute difference between the actual and the predicted measure divided by the actual measure and multiplied by 100 [60]. Furthermore, RMSE was calculated as the square root of the average of the squared differences between predicted and observed values [61].

Finally, to determine any significant differences between the predicted and measured VO_2_max, we used statistical tests, specifically the paired 1-tailed *t* test. We tested the assumption of normally distributed data using the Anderson-Darling test (*P*=.65). Furthermore, we calculate the effect size using Hedges *g*, taking the sample size into account [62], with a value of 0.2 representing a small, 0.5 a medium, and 0.8 a large effect size [63].

### Data Analysis and Fitness Level Categorization

In the first step, we analyzed the entire data set to assess the overall performance of the Apple Watch. Additionally, we aimed to get better insights regarding its performance across varying user fitness levels. To achieve this, participants were categorized into 3 groups based on their reference VO_2_max obtained from the laboratory setting. Hence, based on the fitness categories outlined by the Fitness Registry and the Importance of Exercise National Database [64], participants were divided into poor, good, and excellent fitness levels, allowing us a more nuanced investigation of the Apple Watch’s estimations.

## Results

### Participants

Out of the 30 (14 female and 16 male) initially recruited participants, 6 participants withdrew from the study before the first session due to health and personal reasons. Additionally, after the initial session, 4 participants were deemed ineligible for the study due to health concerns and recommendations from their respective health care providers, and 1 participant did not attend the second session due to personal reasons.

A total of 19 participants successfully completed the initial session in the laboratory setting, which involved a cycle test until subjective exhaustion and metabolic gas analysis, followed by the second session including an outdoor running test. Among the participants, 7 participants were female (mean age 28.86, SD 10.48 years; mean BMI 23.09, SD 2.31 kg/m^2^) and 12 participants were male (mean age 28.17, SD 12.40 years; mean BMI 23.76, SD 3.99 kg/m^2^). Participant characteristics are further detailed in Table 1.

**Table 1:** Participant characteristics.

	Male (n=12, 63%), mean (SD)	Female (n=7, 37%), mean (SD)	Total (n=19), mean (SD)
Age (in years)	28.17 (12.40)	28.71 (10.63)	28.37 (11.48)
BMI (kg/m^2^)	23.92 (3.79)	23.04 (2.11)	23.60 (3.23)

### Limit of Agreement

**Figure 1:**
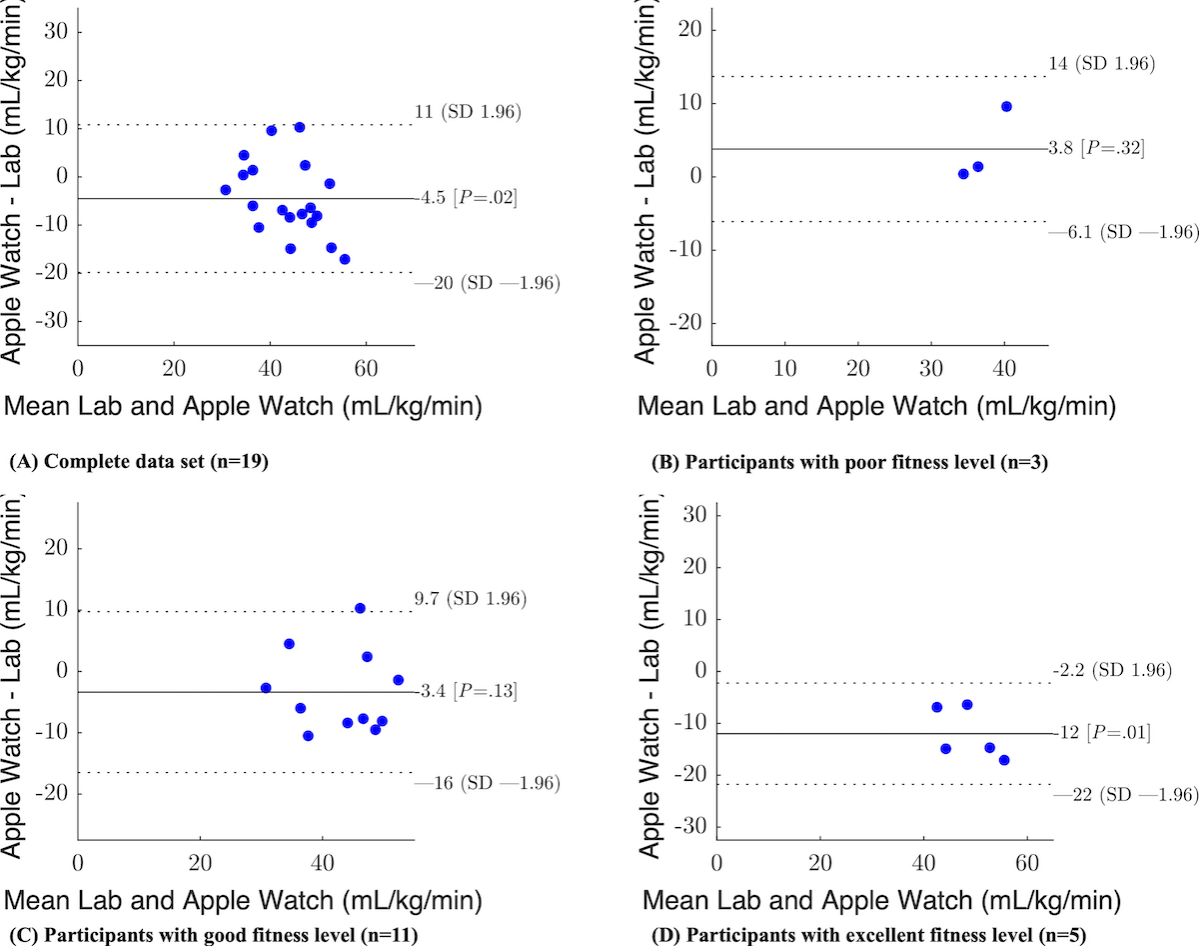
Bland-Altman plot of mean (x-axis) and difference (y-axis) between measured VO2max in the laboratory and predicted VO2max from the Apple Watch. The solid line represents the mean difference and the dashed lines present the 95% limit of agreement.

The detailed results are presented in Table 2. The mean VO_2_max determined in the laboratory setting was 45.88 (SD 9.42) mL/kg/minute, ranging from 32 to 64 mL/kg/minute. Furthermore, the mean estimated VO_2_max from the Apple Watch was 41.37 (SD 6.50) mL/kg/minute, ranging from 29 to 52 mL/kg/minute. Our analysis revealed that the measured VO_2_max is significantly higher than the predicted value from the Apple Watch (t_18_=2.51; *P*=.01) with a medium effect size (Hedges *g*=0.53). These findings are consistent with observations from the Bland-Altman plot (Figure 1A), showing an overall underestimation of VO_2_max by the Apple Watch. Specifically, the mean difference (bias) between the laboratory value and the estimated VO_2_max value from the Apple Watch is –4.51 (SD 7.82) mL/kg/minute. Although all data points fall within the limits of agreement, indicating “good agreement” between the 2 methods, the ICC(2,1) of 0.47 (95% CI 0.06-0.75) suggests only poor to moderate reliability.

**Table 2:** Descriptive examination of the differences between the measured and predicted VO2max.

Fitness level^a^	Participant pool (n=19), n (%)	VO_2_max—Lab^b^ (mL/kg/minute), mean (SD)	VO_2_max—Apple Watch^c^ (mL/kg/minute), mean (SD)	VO_2_max delta^d^ (mL/kg/minute), mean (SD)	MAPE^e^ (%)	RMSE^f^ (mL/kg/minute)	ICC (2,1)^g^ ICC (95% CI)
Poor	3 (16)	35.13 (.81)	38.93 (5.48)	3.8 (5.05)	10.71	5.61	0.14 (–0.61 to 0.96)
Good	11 (58)	44.81 (7.97)	41.44 (7.70)	–3.37 (6.69)	14.59	7.22	0.60 (0.09 to 0.87)
Excellent	5 (26)	54.70 (7.28)	42.70 (4.46)	–12 (4.98)	21.47	12.80	0.23 (–0.07 to 0.79)
Combined	19 (100)	45.88 (9.42)	41.37 (6.5)	–4.51 (7.82)	15.79	8.85	0.47 (0.06 to 0.75)

^a^Categorized according to sex and age based on the Fitness Registry and the Importance of Exercise National Database [64] criteria.

^b^VO_2_max—Lab: measured VO_2_max in the laboratory.

^c^VO_2_max—Apple Watch: estimated VO_2_max from the Apple Watch.

^d^VO_2_max delta: Apple Watch estimate versus laboratory measurement.

^e^MAPE: mean absolute percentage error.

^f^RMSE: root mean square error.

^g^ICC (2,1): intraclass correlation coefficient.

We furthermore analyzed the limit of agreement for participants with lower and higher fitness levels. When the participants were split into groups of poor (n=3), good (n=11), and excellent (n=5) fitness levels, the smartwatch showed a bias of mean 3.80 (SD 5.05) mL/kg/minute, mean –3.37 (SD 6.69) mL/kg/minute, and mean –12.00 (SD 4.98) mL/kg/minute, respectively. As depicted in Figures 1B-1D, the Apple Watch tends to overestimate VO_2_max for participants with a poor fitness level while underestimating it for those with a higher fitness level. Moreover, the ICC for poor and excellent fitness levels was 0.14 and 0.23, respectively, indicating poor reliability. Only for participants with good (n=11) fitness levels, an ICC(2,1) of 0.60 indicates moderate reliability. However, it is important to highlight the limitations associated with interpreting the results for subgroups due to the small sample size.

### Error Between Predicted and Actual VO2max

The MAPE in the cohort of all participants (n=19) was 15.78%, with an RMSE of 8.85 mL/kg/minute. Upon dividing the VO_2_max values into categories based on poor, good, and excellent fitness levels, the smartwatch showed MAPEs of 10.71%, 14.59%, and 21.47%, respectively. Regarding RMSE, the smartwatch showed values of 5.61, 7.22, and 12.80 mL/kg/minute for participants with poor, good, and excellent fitness levels, respectively. However, as already mentioned before, it is important to emphasize the limitation in interpreting results for subgroups due to the limited sample size.

## Discussion

### Principal Results

The purpose of this study was to assess the accuracy of the VO_2_max estimation of the Apple Watch Series 7. Other validation studies using the Apple Watch focused on evaluating the accuracy of measuring oxygen consumption reserve [41], HR [38,39], HR variability [40], or energy expenditure [37]. To the best of our knowledge, this is the first study validating the VO_2_max using the Apple Watch, aside from Apple’s validation study [42].

Overall, our findings reveal a significant underestimation of the estimated VO_2_max value from the Apple Watch (t_18_=2.51; *P*=.01; bias: mean –4.51, SD 7.82 mL/kg/minute; Hedges *g*=0.53). These results deviate from the original validation study by Apple [42], which reported a smaller bias of mean 1.2 (SD 4.4) mL/kg/minute and mean 1.4 (SD 4.7) mL/kg/minute for the design and validation groups, respectively. However, it is important to acknowledge that our VO_2_max value from the Apple Watch was obtained after only 1 outdoor walking and running session. According to Apple’s explanation, increasing the number of outdoor workouts enhances the accuracy of the VO_2_max estimate [42]. In contrast to our study, Apple’s validation study was designed as a longitudinal study, extending over an average of 441 days for the design group and 390 days for the validation group. The researchers computed the mean and SD for differences between the last estimated VO_2_max from the Apple Watch and the mean VO_2_max value determined in up to 6 maximal or submaximal cardiopulmonary exercise tests while wearing the Apple Watch Series 4. However, it remains unclear how exactly the cardiopulmonary exercise test was conducted. Therefore, a direct comparison of our results with theirs is not feasible as they estimated VO_2_max from multiple workouts. It is plausible that our results would show also a smaller error if the participants in our study wore the watch for a longer duration. Apple’s statement that the VO_2_max estimation by the Apple Watch is accurate and reliable compared to conventional methods of VO_2_max measurement [42] can therefore not be contradicted on the basis of the available findings.

Our findings regarding intraclass correlation reveal that ICC(2,1)=0.47, indicating relatively poor reliability, as outlined in reference [59]. Upon excluding participants with poor and excellent fitness levels and focusing solely on those with good fitness levels, we observed an improved ICC(2,1) value of 0.60, suggesting moderate reliability. These results underscore the influence of fitness levels on the reliability of VO_2_max estimation through the Apple Watch. The validation study conducted by Apple calculating ICC(A,1), yielded values of 0.89 and 0.86 for the design and validation groups, respectively, indicative of good reliability [42]. Notably, Apple’s evaluation involved assessing absolute agreement per participant by comparing the last valid VO_2_max estimate with the value estimated at least 28 days prior. This methodology differs from our approach, where we aimed to evaluate the reliability between laboratory-measured values and Apple Watch estimates without a significant time gap.

### Comparison With Prior Work

There is no standardized threshold for high or low MAPE, but we consider an error below 5% to be a good indicator for an accurate measurement. Regarding our results from the Apple Watch, we can conclude that regardless of the fitness level of the participants, the MAPE exceeded 10%. Unfortunately, related studies do not consistently report MAPE values. Nevertheless, 1 study using Polar [30] showed MAPE values above 10% (specifically 13.2%). In addition, studies with Fitbit devices showed MAPE around 10% [27,65]. Conversely, studies on Garmin devices [25,30,33,35], using algorithms developed by Firstbeat Technologies [15], consistently reported MAPE values well below 10%, highlighting their superior accuracy compared to other smartwatches.

We furthermore attempted to compare our results on ICC with those of other studies. Since not all studies provided comprehensive information regarding ICC forms used, making direct comparisons proved to be challenging. Nevertheless, studies on the Garmin Watch have indicated high reliability, with ICC(2,1)=0.87 [29] or ICC(3,1)=0.94 [35], although it is important to note that the latter study validated the estimation of VO_2_ peak rather than VO_2_max.

In terms of fitness levels, this study aligns with findings from related research using various smartwatches. Consistent with observations from references [30,31,33-35,65], our results suggest a tendency for the Apple Watch to overestimate VO_2_max values among users with poor fitness levels (mean 3.80, SD 5.05 mL/kg/minute) and underestimate them among those with higher fitness levels (mean –3.37, SD 6.69 mL/kg/minute and mean –12.00, SD 4.98 mL/kg/minute for good and excellent fitness levels, respectively). However, it should be noted that this study involved a relatively small sample size, and classifying participants based on their fitness levels further reduced the sample size in each group (n=3 for participants with lower fitness, n=11 for those with good fitness, and n=5 for those with excellent fitness). Despite this limitation, our findings suggest that the Apple Watch may provide more accurate VO_2_max estimates for users with poor or good fitness levels. This conclusion is further supported by MAPE, which shows a smaller error for users with poorer fitness levels while the error increases in participants with higher fitness levels (see also Table 2). This could be attributed to the potential influence of fitness levels on the accuracy of physiological measurements obtained through wearable devices. Nonetheless, further research with larger sample sizes is necessary to validate and elucidate these observations. Such investigations could shed light on the factors influencing the performance of wearable devices in estimating VO_2_max across various fitness levels, thereby enhancing our understanding of their use in health and fitness monitoring.

### Limitations

The major limitation of this study is the small sample size. Although we aimed to recruit at least 30 participants, we ultimately obtained complete data from only 19 participants. To address this limitation, we reported effect sizes alongside our statistical tests, ensuring that our results remain reliable despite the smaller sample size. Nevertheless, further studies with larger and more varied populations are recommended to build on these findings and enhance the statistical power of the conclusions. It would also be beneficial to extend the duration during which participants consistently wear a smartwatch, as we believe that longer wear periods may enhance the accuracy of VO_2_max estimation by the Apple Watch.

Although VO_2_max measurement is considered the gold standard among sports medicine professionals for determining an individual’s fitness level, prior research has suggested that VO_2_max is constrained by the variability in an individual’s effort and is highly reliant on VO_2_max extent to which participants are properly motivated to achieve their true maximum [66]. Furthermore, as VO_2_max criteria are not standardized, there is some uncertainty regarding whether the true VO_2_max has actually been attained and if a maximum effort has been exerted [67]. To address these concerns, Edvardsen et al [68] proposed revised termination criteria for VO_2_max tests that consider sex and age. Furthermore, as the true VO_2_max value can differ, depending on whether the cardiopulmonary exercise testing was done on a treadmill or cycle ergometer, it would be important to use both tests independently to achieve optimal fitness assessment [69]. Nevertheless, varying termination criteria, testing methodologies, and participant populations across studies continue to pose challenges [67]. Despite these challenges, our aim involved making selective comparisons between our study and related research, diligently acknowledging the notable differences between the studies.

Another limitation we encountered was related to calibration error. Our attempt to compare the approximate prediction method of the Apple Watch with a gas analyzer was conducted using a graded exercise test until subjective exhaustion, potentially leading to an underestimation of the true VO_2_max value. Noonan and Dean [70] outlined the advantages of submaximal exercise tests over maximal exercise tests, citing factors such as requirements for trained personnel and safety concerns. They conclude that submaximal exercise tests are reliable if an appropriate protocol is selected and the protocol is followed. However, it is crucial to note the potential influence of different protocols or increased participant motivation, as these factors could impact the measured VO_2_max.

An additional limitation of our study is the lack of medical equipment. Ideally, we would have conducted periodic blood samples to measure the lactate threshold, allowing us to detect the point when the participant’s respiratory system attained its maximum capacity. The lactate concentration in blood is a valuable metric to monitor because an increase in blood lactate indicates a transition from aerobic to anaerobic exercise, suggesting that the body has surpassed its capacity for oxygen uptake to supply the muscles adequately [71]. Unfortunately, due to the unavailability of suitable equipment and the lack of medical professionals capable of carrying out such data collection, we were unable to include blood lactate as a termination criterion in our study. Additionally, it would have been ideal to monitor the volume of carbon dioxide produced; however, this capability is not provided by the VO2 Master Analyzer.

### Conclusions

Overall, the Apple Watch Series 7 underestimated VO_2_max compared to the values obtained using the gold standard assessment methods within a laboratory setting. This underestimation was even pronounced in participants with very high fitness levels. On the contrary, VO_2_max values were overestimated by the Apple watch in participants with comparably low fitness levels. These findings highlight the importance of calibrating consumer-grade fitness trackers for greater accuracy across a diverse range of fitness levels. As consumer-grade technology continues to evolve, there is an opportunity for ongoing research and development to close the gap between the accuracy of portable devices and laboratory-grade equipment. This would not only enhance individual training and health monitoring but could also expand the use of such wearables in professional sports and clinical settings.

## Abbreviations

HR: heart rate
ICC: intraclass correlation coefficient
MAPE: mean absolute percentage error
RMSE: root-mean-square error
VO2max: maximum oxygen uptake
